# Establishment and characterization of *adap1* ‐deficient zebrafish

**DOI:** 10.1111/dgd.70004

**Published:** 2025-03-15

**Authors:** Atsuo Kawahara, Sakyo Yasojima, Junko Koiwa, Saori Fujimaki, Hiroaki Ito, Mamiko Yamada, Kenjiro Kosaki, Yuhei Nishimura

**Affiliations:** ^1^ Laboratory for Developmental Biology, Center for Medical Education and Sciences, Graduate School of Medical Science University of Yamanashi Yamanashi Japan; ^2^ Department of Integrative Pharmacology Mie University Graduate School of Medicine Tsu Japan; ^3^ Center for Medical Genetics Keio University School of Medicine Tokyo Japan

**Keywords:** *adap1*, locomotor activity, morphogenesis, social behavior, zebrafish

## Abstract

The *adap1* (ADP‐ribosylation factor GTPase‐activating protein [ArfGAP] with dual pleckstrin homology [PH] domains 1) gene is predominantly expressed in the mouse brain and is important in neural differentiation and development. However, the functions of *adap1* in morphogenesis, locomotor activity, and behaviors in vertebrates are not fully understood. Whole‐mount in situ hybridization (WISH) analysis revealed that *adap1* was widely expressed in the zebrafish brain, including the forebrain, midbrain, and hindbrain, during early embryogenesis. To investigate the physiological function of the *adap1* gene, we generated zebrafish *adap1* mutants harboring frameshift mutations around codon 120 of *adap1*. The *adap1* mutants containing homozygous mutant alleles exhibited no apparent morphological abnormalities at 1 day postfertilization (dpf), and the spontaneous coiling and touch response of the *adap1* mutants were comparable to those of the wild‐type fish. In addition, the expression of neural genes, such as *emx1*, *mbx*, and *huC*, was comparable between the wild‐type fish and the *adap1* mutants at 1 dpf. The *adap1* mutants grew to adulthood without exhibiting any apparent swimming defects. The adult *adap1* mutants spent more time than the wild type in the center region of the open field test. In the social behavior test, zebrafish containing the *adap1* mutant alleles spent more time than the wild type in the regions near the chambers where novel conspecifics swam. These results imply the involvement of the *adap1* gene in regulating approach behavior to visual cues from conspecifics.

## Graphical abstract

To examine the physiological functions of the *adap1* gene, we have established zebrafish *adap1* mutants lacking functional PH1 and PH2 domains. The *adap1* mutants showed no apparent morphological abnormalities and grew to adulthood. We found that the adult *adap1* mutants spent more time than the wild type in the regions near the chambers where novel conspecifics swam, suggesting the involvement of the *adap1* gene in the social behavior.

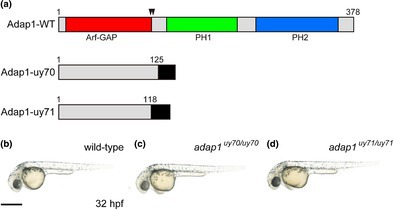

## 1 INTRODUCTION

*adap1*/Centaurin‐α1 was originally identified as a protein that binds to an affinity matrix composed of phosphatidylinositol (3,4,5)‐triphosphate (PIP_3_) in rat brains (Venkateswarlu et al., 1999). *adap1* includes an ArfGAP domain in the N‐termus and two PH domains in the C‐terminus. The PH domains of *adap1* can bind to PIP_3_ and inositol 1,3,4,5‐tetrakisphosphate (IP_4_), suggesting that *adap1* is involved in mediating delivery of the second messenger PIP_3_ to the axon tip (Duellberg et al., 2021). The ArfGAP domain of *adap1* is believed to catalyze the GTP hydrolysis of ARF6, presumably leading to the regulation of dendrite branching by controlling ARF6 activity (Venkateswarlu et al., 2004). Inactivation of the *adap1* gene in mice does not influence gross brain morphology (Szatmari et al., 2021). *adap1* deficiency results in a progressive increase in dendritic spine density in the hippocampus and an improvement in hippocampus‐dependent memory (Szatmari et al., 2021). These results from *adap1* knockout (KO) mice suggest that *adap1* functions as a negative regulator of dendritic spine density in the hippocampus and hippocampus‐dependent spatial memory formation. Thus, *adap1* plays important roles in neural development and neural function. However, the functions of *adap1* in morphogenesis, locomotor activity, and social behavior remain unknown.

The zebrafish is an ideal vertebrate model for genetic analysis (Hisano et al., 2014). Zebrafish embryos, which rapidly develop outside the mother's body, are transparent, and most organs start to function within several days after fertilization. Thus, the developmental function of the gene of interest can be assessed by loss‐of‐function analysis during zebrafish early embryogenesis. Recently, zebrafish have also been widely used as a vertebrate model to study social behavior (Geng & Peterson, 2019). Accumulating evidence suggests that zebrafish are suitable model organisms for human neurodevelopmental disorders (Abreu et al., 2020; Suzuki et al., 2022).

In the zebrafish genome, we identified the *adap1* gene that potentially possessed high similarity to the human *adap1* gene. To examine the functional relationship between human *adap1* and zebrafish *adap1*, we isolated the zebrafish *adap1* gene from a wild‐type cDNA library. We next examined the expression of the *adap1* gene during early zebrafish embryogenesis and found that *adap1* was widely expressed in the brain, including the forebrain, midbrain, and hindbrain, during early embryogenesis. In this study, we established two *adap1* zebrafish mutants and investigated the physiological function of *adap1* via phenotypic analysis.

## 2 MATERIALS AND METHODS

### 2.1 Isolation of the *adap1* gene from zebrafish

Both *adap1*‐N (PCR product length, 642 bp) and *adap1*‐C (PCR product length, 976 bp), which contain overlapping sequences, were isolated from a 24 hr postfertilization (hpf) zebrafish cDNA library by using the following oligonucleotide primers: *adap1*‐BamHI‐F1, 5′‐CGGGATCCATGATGGAGAACGAGCCGGAG‐3′ (underline, start codon), and *adap1*‐EcoRI‐R2, 5′‐GGAATTCGTGCTGTTGTCCTTCAGGTATG‐3′ for *adap1*‐N; *adap1*‐BamHI‐F2, 5′‐CGGGATCCGGTGTGAAGTCGGTCCTGCAGG‐3′, and *adap1*‐EcoRI‐R1, 5′‐GGAATTCTCAAGGCTTGTGCTTGAAATG‐3′ (underline, stop codon) for *adap1*‐C. After *Bam*HI and *Eco*RI restriction enzyme digestion, the resulting DNA fragments were inserted into the *Bam*HI‐*Eco*RI‐cleaved pCS2P vector. The sequence of the isolated *adap1* gene was determined by sequence analysis. The DNA Data Bank of Japan (DDBJ) accession number of the zebrafish *adap1* gene is LC781536.

### 2.2 Phylogenic analysis of *adap* family genes

For phylogenic tree analysis, Adap protein sequences, such as human *adap1* (acc. no.: NP_006860.2), human ADAP2 (Acc. No.: NP_001333641.1), zebrafish ADAP2 (acc. no.: XP_021329475.1), and an uncharacterized gene product zgc_92360 (acc. no.: NP_001002715.1), were obtained from National Center for Biotechnology Information (NCBI) protein database. The above sequences and our isolated *adap1* (LC781536) were aligned with MAFFT and the tree was constructed with neighbor joining (NJ) algorithm (Kuraku et al., 2013).

### 2.3 Synthetic crRNA and tracrRNA, recombinant Cas9 protein, and microinjection

To induce insertion/deletion (indel) mutations in the *adap1* genomic locus, we used a ready‐to‐use CRISPR–Cas9 system composed of CRISPR RNA (crRNA), *trans‐*activating crRNA (tracrRNA), and recombinant Cas9 protein (Kotani et al., 2015). The synthetic crRNAs (Table S1), and tracrRNA and recombinant Cas9 protein were obtained from Integrated DNA Technology, Inc. (IDT). Synthetic *adap1*‐crRNA1 (25 pg), *adap1*‐crRNA2 (25 pg), and tracrRNA (100 pg) were co‐injected with recombinant Cas9 protein (1 ng) into zebrafish embryos at the one‐cell stage.

### 2.4 Establishment of *adap1*–deficient mutants

F0 embryos injected with *adap1*‐crRNAs, tracrRNA and Cas9 protein developed into adult (3 months postfertilization, 3 mpf). We found three F0 founders that produced several insertion and/or deletion (indel) mutations in the targeted *adap1* locus, when individual founder fish was crossed to wild‐type fish. We finally obtained two mutant alleles (*uy70* and *uy71*) for the *adap1* locus as described below. We usually maintained heterozygous *adap1* ^*uy70/WT*^ and *adap1* ^*uy71/WT*^ fish. To obtain homozygous *adap1* mutants, heterozygous adult *adap1* mutants were crossed. Subsequently, the genotype of individual fish was determined by a heteroduplex mobility assay (HMA) using genomic DNA from the fin clip of growing fish (Ota et al., 2014).

### 2.5 Genotyping of the *adap1* locus and genomic sequencing

The *adap1* ^*uy70*^ allele carries a deletion of bp 378–381 in the *adap1* coding region (−4 bp), and the *adap1* ^*uy71*^ allele carries deletions of bp 356–358 and bp 376–379 in the *adap1* coding region (total −7 bp). To isolate the genomic DNA, the individual embryos or fins were incubated in 108 μl of 50 mM NaOH at 98°C for 10 min. Subsequently, 12 μl of 1 M Tris–HCl (pH 8.0) was added to the solution. Genomic fragments for the targeted genomic locus were amplified by PCR with PrimeTaq (Primetech) using locus‐specific primers (Table S2). The PCR conditions were as follows: 95°C for 2 min, followed by 40 cycles of 98°C for 10 s, 55°C for 30 s, and 72°C for 30 s. The resulting PCR amplicons were electrophoresed on a 12.5% polyacrylamide gel for the HMA. The resulting PCR fragments were subcloned and inserted into pGEM‐T Easy vector (Promega), and the genomic sequences were determined by sequence analysis.

### 2.6 Time lapse analysis of zebrafish behavior

Embryonic behaviors were observed using a stereomicroscope (ZEISS Axio Zoom V16). Spontaneous coiling and touch responses elicited by mechanosensory stimulation delivered to the trunk with an eyelash were analyzed by a high‐speed camera (HAS‐U1, DITECT Co. Ltd.). The swimming behaviors of adult zebrafish were analyzed by a digital camera (D5500, Nikon).

### 2.7 Whole‐mount in situ hybridization (WISH)

As previously described (Kawahara et al., 2009), we examined the expression of the *adap1*, *emx1*, *mbx*, *islet1*, *oligo2*, *gfap*, and *huC* genes by WISH analysis. The embryos at different developmental stages were fixed by 4% paraformaldehyde (PFA). The embryos at 48 hpf and at 72 hpf were treated with protenase K (final concentration; 10 μg/ml) for 30 and 60 min, respectively. Zebrafish embryos stained with the indicated digoxygenin (DIG)‐labeled RNA probe were incubated with alkaline phosphatase‐conjugated anti‐DIG antibody. To visualize the RNA probe recognized by the anti‐DIG antibody, the samples were subsequently incubated with BM Purple (Roche) as the substrate. After three washes with PBST, the samples were fixed in 4% PFA.

### 2.8 Behavioral analysis of adult zebrafish

The tests for zebrafish behavior were used with some modifications as described below (Ribeiro et al., 2020). For the open field and the social behavior tests, three clear plastic chambers were tandemly aligned. The width, depth, and height of each chamber were 8.4, 8.4, and 5 cm, respectively. Each chamber was filled with 130 ml of water taken from the housing system. A wild‐type or *adap1* mutant zebrafish was placed in the middle chamber (chamber II). In the social behavior test, one albino or Riken Wild (RW) zebrafish was placed in a side chamber (chamber I), and three albino or RW zebrafish were placed in the other side chamber (chamber III). We used both female and male zebrafish at 3, 4, or 7 month‐post‐fertilization in chamber II. After 5 min of acclimation, the behavior of the zebrafish was recorded for using an iPod touch (Apple, CA, USA), which was set laterally of the chamber for the novel tank diving test and upward of the chambers for the open field and the social behavior tests. We recorded individual zebrafish behavior for 20 min in the open field and the social behavior tests. The movie files were converted to files of 25 frames per s using FFmpeg (https://ffmpeg.org/) and input into UMATracker (Yamanaka & Takeuchi, 2018) to analyze the trajectory of the zebrafish. Heat maps were generated using the trajectory data of all zebrafish in the indicated group using the libraries in the R framework, including ggplot2 (Wickham, 2016) and tidyverse (Wickham et al., 2019). We measured the distance of how far zebrafish moved in the chamber. For the open field test (Liu et al., 2018), we measured the time the zebrafish were localized in area 1, a 4.5 cm square at the center of chamber II, and area 2, the region of chamber II excluding area 1. For the social behavior test (Kim et al., 2017), we measured the time the zebrafish were localized in area 3, the region near chamber I; area 5, the region near chamber III; and area 4 of chamber II, the region of chamber II excluding areas 3 and 5. The mean distance moved in the chamber or time spent in these areas in each minute was plotted for each group with the standard error. Generalized linear mixed model was used to assess the difference between mutant and wild‐type fish using lme4, lmerTest, and emmeans in the R framework. A *p*‐value <0.05 was considered to indicate statistical significance.

## 3 RESULTS

### 3.1 Isolation of zebrafish *adap1* and developmental *adap1* expression during zebrafish early embryogenesis

Human *adap1*, which consists of 378 amino acids, possesses three functional domains, the ArfGAP, PH1, and PH2 domains (Stricker & Reiser, 2014). In the zebrafish genome, we found a potential *adap1* gene that had significant homology to the human *adap1* gene. We isolated the zebrafish *adap1* gene from the zebrafish cDNA library (LC781536); the predicted amino acid sequence of *adap1* contained the ArfGAP, PH1, and PH2 domains, and had 69.71% amino acid identity to human *adap1* (Supplementary Figure S1). Phylogenic analysis of zebrafish and human Adap family proteins suggest that zebrafish *adap1* and human *adap1* are highly homologous (Supplementary Figure S2). This high homology between human *adap1* and zebrafish *adap1* suggests that they are orthologue of each other because other Adap family products show less homology (Venturin et al., 2014).

**FIGURE 1:**
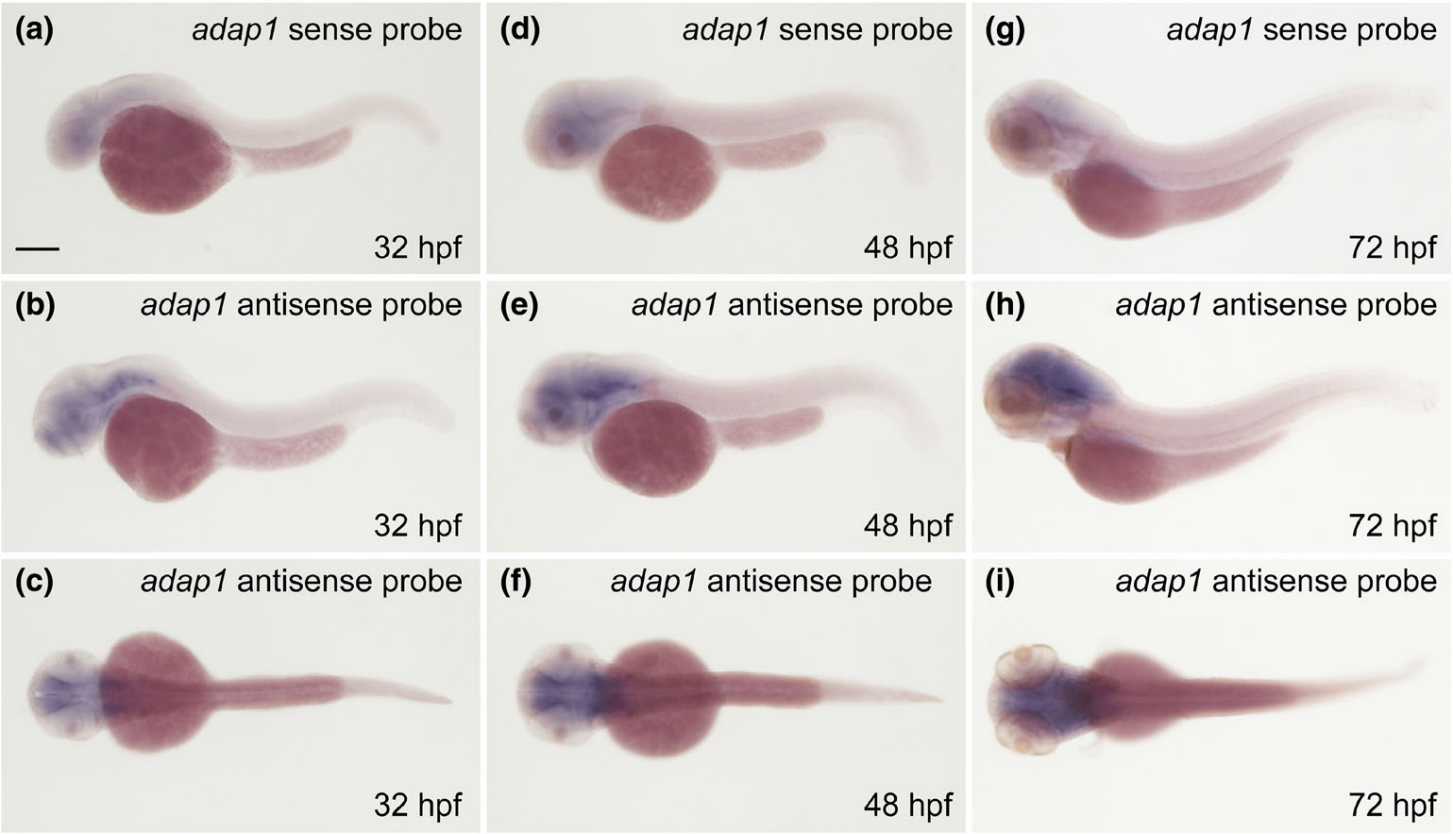
Expression of *adap1* during early embryogenesis. (a–c) Embryos stained with the *adap1* sense probe (a: Lateral view) and the *adap1* antisense probe (b: Lateral view, c: Dorsal view) at 32 hpf. (d–f) Embryos stained with the *adap1* sense probe (d: Lateral view) and the *adap1* antisense probe (e: Lateral view, f: Dorsal view) at 48 hpf. (g–i) Embryos stained with the *adap1* sense probe (g: Lateral view) and the *adap1* antisense probe (h: Lateral view, i: Dorsal view) at 72 hpf. Expression of *adap1* was widely detected in the forebrain, midbrain and hindbrain at 32 hpf. Expression of *adap1* in the midbrain, and hindbrain was maintained at 48 hpf and 72 hpf. Scale bar, 200 μm (a).

Because the developmental expression of the zebrafish *adap1* gene remains unknown, we first examined *adap1* expression during early embryogenesis using WISH analysis. Expression of *adap1*, which was detected by staining with the antisense *adap1* DIG RNA probe but not sense *adap1* DIG RNA probe, was specifically observed in the forebrain, midbrain and hindbrain at 32 hpf (Figure 1). The expression of *adap1* in the midbrain and hindbrain was maintained at 48 hpf and 72 hpf, suggesting that the *adap1* gene is involved in the establishment of the central nervous system in zebrafish.

### 3.2 Establishment of two *adap1* mutants and spontaneous coiling and touch responses in the *adap1* mutants

**FIGURE 2:**
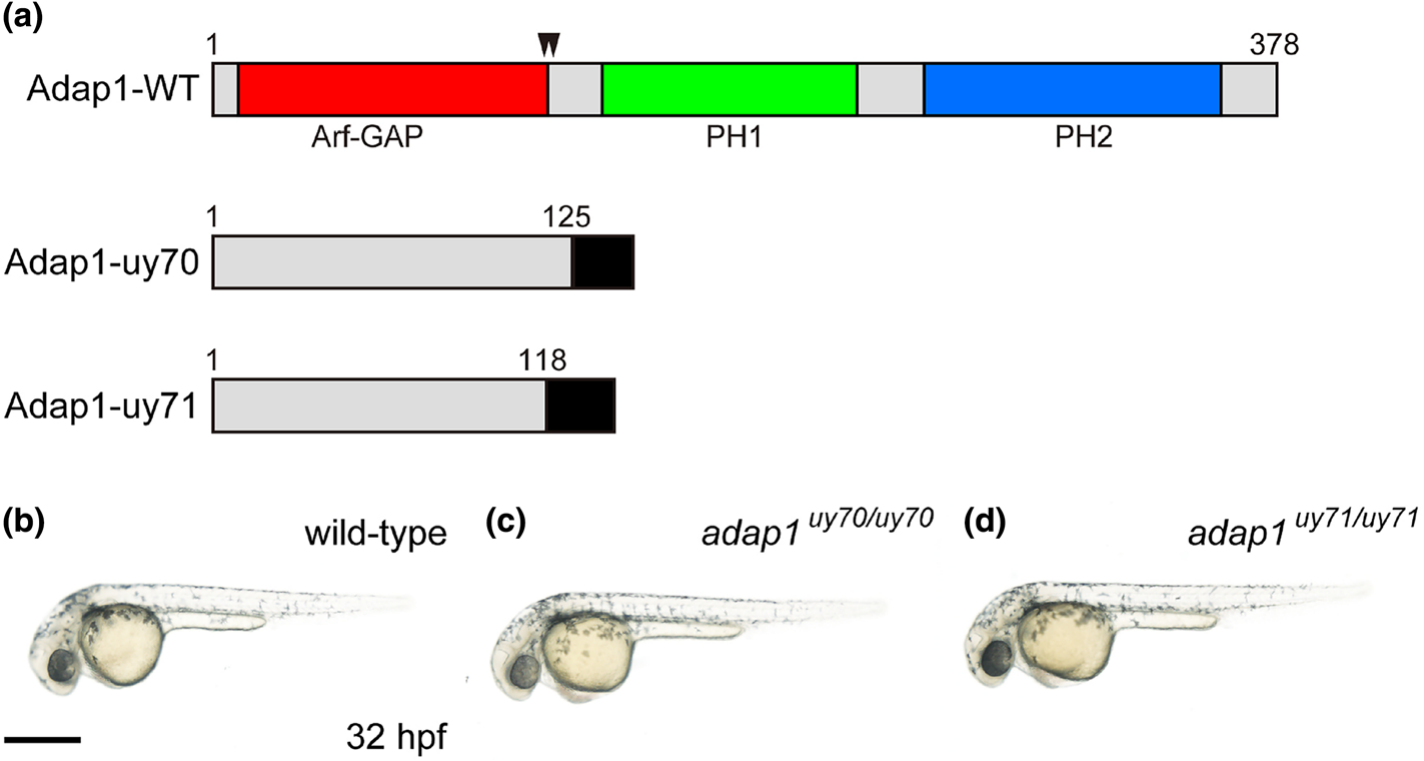
Establishment of *adap1* mutants. (a) Molecular structure of *adap1*. The ArfGAP, pleckstrin homology 1 (PH1) and PH2 domains in the Adap1 protein are indicated by the red, green, and blue rectangles, respectively. The *adap1*‐uy70 and *adap1*‐uy71 mutants contained premature stop codons after 24 missense amino acids starting at amino acid 125 and after 30 missense amino acids starting at amino acid 118, respectively. (b–d) Morphology of the wild‐type (b), *adap1* ^*uy70/uy70*^ mutant (c), and *adap1* ^*uy71/uy71*^ mutant (d) zebrafish. Scale bar, 500 μm (b). Genotyping of individual embryos was performed by genomic PCR.

To examine the physiological function of the *adap1* gene, we designed *adap1* crRNAs (*adap1*‐crRNA1 and *adap1*‐crRNA2) targeting codon 120 in *adap1* to introduce frameshift mutations (Supplementary Table S1). We generated two *adap1* mutants harboring frameshift mutations that lacked both the PH1 and PH2 domains (Figure 2a and Supplementary Figure S3) and performed a phenotypic analysis of these mutants.

We first investigated the morphology and early locomotor activities of *adap1* mutants during early embryogenesis (McKeown et al., 2009). We found that morphology of the *adap1* mutants (*adap1* ^*uy70/uy70*^; *n* = 7; *adap1* ^*uy71/uy71*^; *n* = 6) was indistinguishable from that of the wild‐type fish (*n* = 9) at 30 hr postfertilization (hpf) (Figure 2b–d). Spontaneous coiling is a slow contraction of the trunk that occurs at approximately 19 hpf (Cui et al., 2005). The *adap1* mutants exhibited normal spontaneous coiling at a frequency (*adap1* ^*uy70/uy70*^; 0.56 ± 0.07 Hz, *n* = 8; *adap1* ^*uy71/uy71*^; 0.54 ± 0.07 Hz, *n* = 8) that was comparable to that of the wild‐type fish (0.57 ± 0.07 Hz, *n* = 7) at 19 hpf (Supplementary Movies [Link], [Link], [Link]). Next, we examined escape and swimming responses to mechanosensory stimulation (Saint‐Amant & Drapeau, 1998). Wild‐type (*n* = 7) and *adap1* mutant (*adap1* ^*uy70/uy70*^; *n* = 8; *adap1* ^*uy71/uy71*^; *n* = 7) fish responded similarly to tactile stimulation, with alternating contractions of the trunk (Supplementary Movies [Link], [Link], [Link]). These results suggested that *adap1* mutants normally develop without apparent morphological or locomotion defects during early embryogenesis.

**FIGURE 3:**
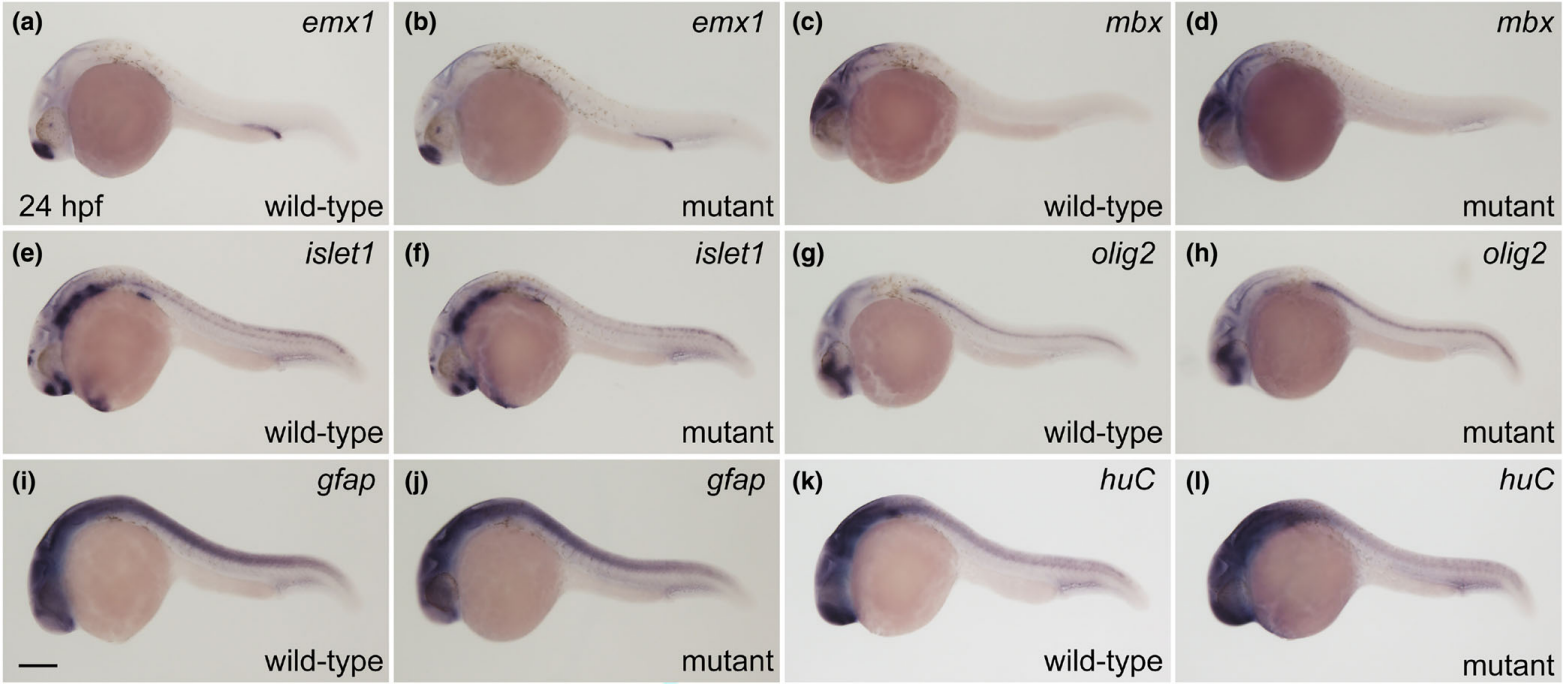
Neural gene expression in wild‐type and *adap1* mutant fish. (a, c, e, g, i, k) Embryos with the *adap1* wild‐type allele at 24 hpf. (b, d, f, h, j, l) Embryos with the *adap1* homozygous mutant alleles at 24 hpf. WISH with *emx1* (a, b), *mbx* (c, d), *islet1* (e, f), *oligo2* (g, h), *gfap* (i, j) and *huC* (k, l). All the images show lateral views, with the anterior to the left. Scale bar, 200 μm (i). The expression of *emx1*, *mbx*, *islet1*, *oligo2*, *gfap*, and *huC* was comparable between the wild‐type and the *adap1* mutant embryos at 24 hpf. Genotyping of individual embryos was performed by genomic PCR.

We also examined the expression patterns of glial and neural genes in the *adap1* mutants containing homozygous mutant alleles using WISH analysis. The forebrain gene *emx1* (wild type: *n* = 7; mutant: *n* = 8), midbrain/hindbrain gene *mbx* (wild type: *n* = 6; mutant: *n* = 8), and glial cell/spinal cord genes *oligo2* (wild type: *n* = 8; mutant: *n* = 8) and *gfap* (wild type: *n* = 10; mutant: *n* = 4) were expressed at comparable levels in the wild‐type and *adap1* mutant models at 24 hpf (Figure 3). Both the motor neuron gene *islet1* (wild‐type: *n* = 5; mutant: *n* = 10) and the neuron gene *huC* (wild type: *n* = 8; mutant: *n* = 8) were comparably expressed in the wild‐type and *adap1* mutant models at 24 hpf (Figure 3).

### 3.3 Behavioral analyses of the *adap1* mutant fish at the adult stage

**FIGURE 4:**
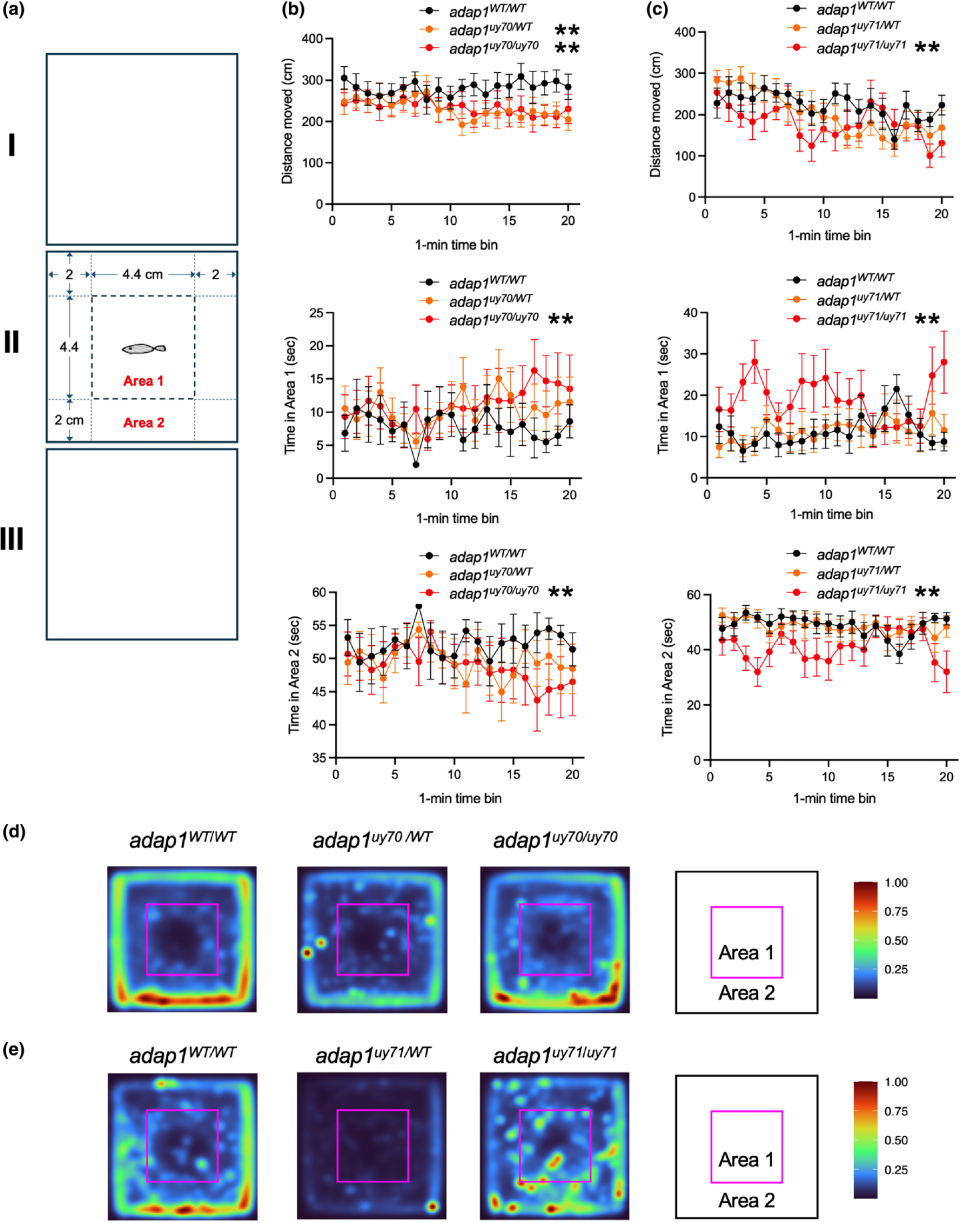
Open field test of wild‐type and *adap1* mutants in the adult stage. (a) Schematic representation of the open field test used in this study. (b and c) The distance moved by zebrafish in chamber II and the time spent in each area (area 1 and 2) for each minute. The distance moved of *adap1* ^*uy70/WT*^, *adap1* ^*uy70/uy70*^, and *adap1* ^*uy71/uy71*^ was shorter than that of wild‐type. The *adap1* ^*uy70/uy70*^ and *adap1* ^*uy71/uy71*^ stayed longer in area 1 and both stayed less time in area 2. The numbers of zebrafish are *Ν* = 11 for WT/WT, *Ν* = 18 for WT/*uy70*, *Ν* = 15 for *uy70*/*uy70*, *Ν* = 10 for WT/WT, *Ν* = 12 for WT/*uy71*, *Ν* = 11 for *uy71*/*uy71*. *p < 0.05, **p < 0.01. (d and e) Heat map of the behavior of wild‐type and *adap1* mutants. The numbers of zebrafish in each group are same in (b and c). A high number of color indicators indicates that zebrafish were frequently located at that position during the 20 min test period.

**FIGURE 5:**
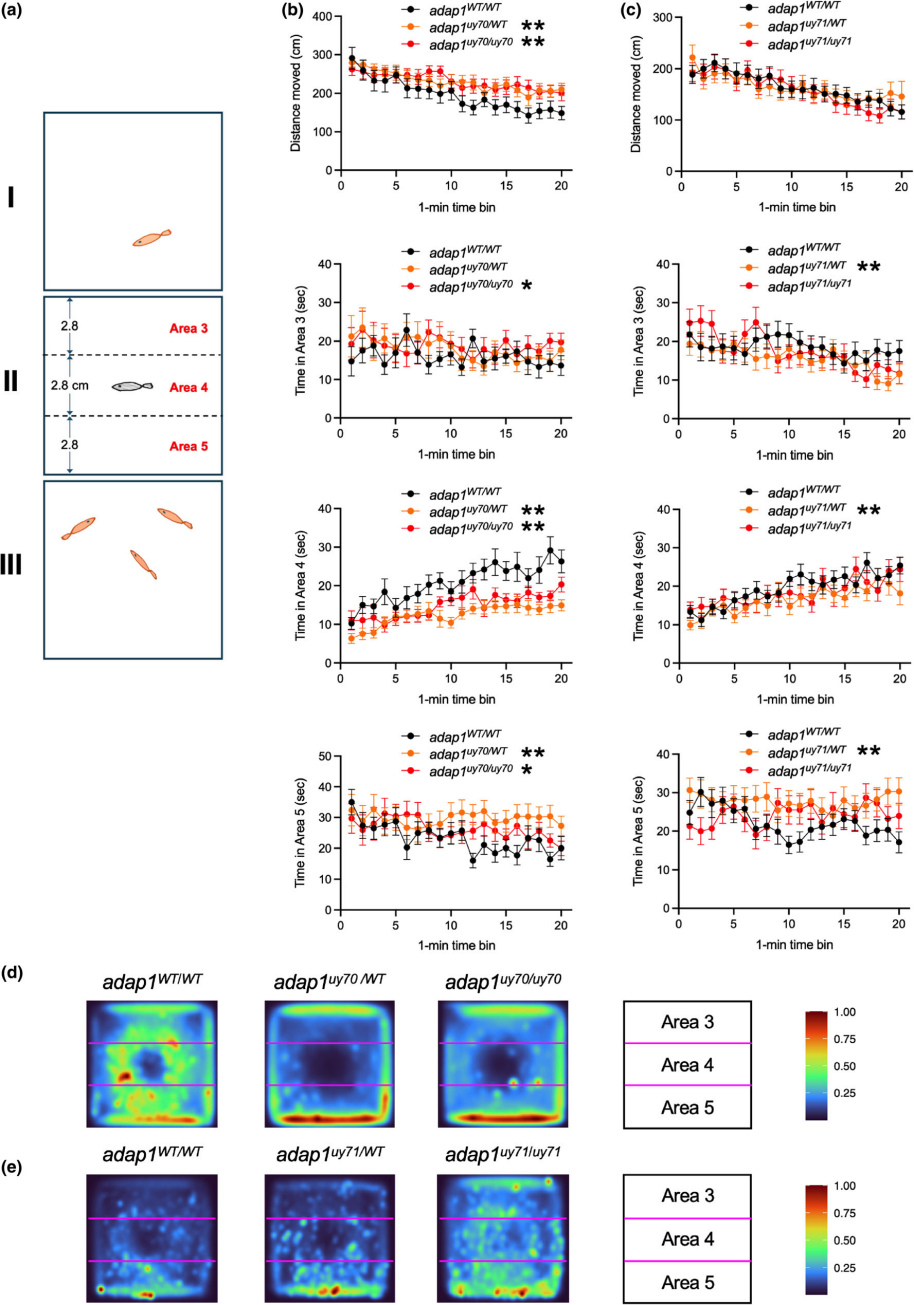
Social behavior analysis of wild‐type and *adap1* mutants in the adult stage. (a) Schematic representation of the social behavior test used in this study. Areas 3 and 5 were set as the region near the stimulus exploration. One and three novel conspecifics (e.g., albino line) were placed in chamber I and III, respectively. (b and c) The distance moved by zebrafish in chamber II and the time spent in each area (areas 3, 4, and 5) for each minute. The *adap1* ^*uy70/WT*^ and *adap1* ^*uy70/uy70*^ swam longer than the wild type (WT). The *adap1* ^*uy70/uy70*^ stayed longer than the wild type in area 3. The *adap1* ^*uy71/WT*^ stayed shorter than the wild type in area 3. The *adap1* ^*uy70/WT*^, *adap1* ^*uy70/uy70*^, and *adap1* ^*uy71/WT*^ stayed shorter than the wild type in area 4. The *adap1* ^*uy70/WT*^, *adap1* ^*uy70/uy70*^, and *adap1* ^*uy71/WT*^ stayed longer than the wild type in area 5. The numbers of zebrafish are *Ν* = 16 for WT/WT, *Ν* = 19 for WT/*uy70*, *Ν* = 17 for *uy70*/*uy70*, *Ν* = 30 for WT/WT, *Ν* = 34 for WT/*uy71*, *Ν* = 29 for *uy71*/*uy71*. *p < 0.05, **p < 0.01. (d and e) Heat map of the behavior of wild‐type and *adap1* mutants. The numbers of zebrafish in each group are same in (b and c). A high number of color indicators indicates that zebrafish were frequently located at that position during the 20 min test period.

We observed that both the *adap1* ^*uy70/uy70*^ and *adap1* ^*uy71/uy71*^ mutants exhibited swimming behaviors comparable to those of the wild‐type fish (Supplementary Movies [Link], [Link], [Link]). Genotyping of offspring from heterozygous mutant intercrosses confirmed the presence of adult *adap1* ^*uy70/uy70*^ and *adap1* ^*u71/uy71*^ (Table 1), indicating the survival of these mutant zebrafish to adulthood. We then examined the behavior of wild‐type and *adap1* mutant fish at the adult stage. Open field test (Figure 4) and social behavior test (Figure 5 and Supplementary Figure S4) were performed.

**TABLE 1:** Numbers of wild‐type (WT), homozygous, and heterozygous *adap1* mutant offspring from genotyping analysis of heterozygous intercrosses at 5 mpf. Alleles of *yu70* and *yu71* contain deletions of four and seven base pairs in the adap1 cording region, respectively.

WT/WT	−4/WT	−4/−4
9	25	11

WT/WT	−7/WT	−7/−7
31	44	26

In the open field test, a wild‐type, heterozygous, or homozygous *adap1* mutant was placed in the middle chambers (chamber II). We analyzed individual zebrafish behavior focusing on the distance moved in chamber II and time spent in areas 1 and 2 (Figure 4a). The *adap1* ^*uy70/uy70*^ and *adap1* ^*uy71/uy71*^ spent longer and shorter than the wild‐type in area 1 and area 2, respectively (Figure 4b,c). The heat maps of zebrafish behavior showed brighter colors for *adap1* mutants in area 1 compared to those of wild‐type (Figure 4d,e), presenting some behavior differences between the *adap1* mutants and wild type.

In the social behavior test, a wild‐type, heterozygous, or homozygous *adap1* mutant was placed in chamber II. One and three novel (Figure 5) or familiar (Supplementary Figure S4) conspecifics were placed in chamber I and III, respectively. We analyzed individual zebrafish behavior in chamber II focusing on the distance moved in chamber II and time spent in areas 3, 4, and 5 (Figure 5a and Supplementary Figure S4a). As shown in Figure 5 and Supplementary Figure S4, the time spent in area 5 of the *adap1* mutants and wild type was longer than those in area 3. This social behavior may be associated with the group preference of zebrafish. In the social behavior test using novel conspecifics, *adap1* ^*uy70/WT*^, *adap1* ^*uy70/uy70*^, and *adap1* ^*uy71/WT*^ spent shorter in areas 4 and spent more time in areas 5 (Figure 5b,c). In the social behavior test using familiar conspecifics, *adap1* ^*uy70/WT*^, *adap1* ^*uy70/uy70*^, *adap1* ^*uy71/WT*^, and *adap1* ^*uy71/uy71*^spent less time in areas 4. whereas *adap1* ^*uy70/WT*^, *adap1* ^*uy70/uy70*^, and *adap1* ^*uy71/WT*^ spent less time in areas 5 (Supplementary Figure S4). The results of above behavioral analyses suggest the *adap1* gene is involved in the regulation of approach behavior to visual cues from conspecifics.

## 4 DISCUSSION

In this study, we isolated the zebrafish *adap1* gene, which is highly homologous to the human *adap1* gene. Because the expression of the zebrafish *adap1* gene was widely detected in the brain, including the forebrain, midbrain, and hindbrain, during early embryogenesis (Figure 1), this gene may be involved in the functional establishment of the central nervous system. We generated two *adap1* zebrafish mutants that possessed frameshift mutations around codon 120 in *adap1* (Figure 2) and lacked both the PH1 and PH2 domains. We observed that the *adap1* mutants developed without apparent morphological or locomotive abnormalities during early embryogenesis. Both the spontaneous coiling and touch response of the *adap1* mutants were comparable to those of the wild‐type fish. Similarly, deficiency of the *adap1* gene in mice does not influence gross brain morphology (Szatmari et al., 2021). In zebrafish, various neural genes, such as *emx1*, *mbx*, *oligo2*, *gfap*, *islet1*, and *huC*, were expressed at comparable levels in the *adap1* mutants (Figure 3). These results suggest that the *adap1* gene is dispensable during early embryogenesis in zebrafish. Another possibility is that other genes in the *adap* family may redundantly compensate for the function of *adap1* in the mutants. Genes in the *adap* family are structurally similar to *the adap1* gene in the zebrafish genome (Venturin et al., 2014).

According to the genotyping of heterozygous intercrosses, *adap1* mutants are viable as adult fish with normal swimming ability, suggesting that *adap1* mutants can survive without obvious developmental defects. Zebrafish are suitable model organisms for studying behaviors associated with neurodevelopmental disorders (Abreu et al., 2020). We found that adult *adap1* mutants spent more time in the center region in the open field test (Figure 4). In the social behavior test, zebrafish containing *adap1* mutant alleles spent more time in the regions near the chamber where novel conspecifics swam (Figure 5). It is not clear why heterozygous *adap1* mutants exhibit some differences in social behavior. One possible explanation is that quantities and/or distribution of the *adap1* products in the brain may affect social behavior. Further analyses are necessary to define the molecular mechanism by which *adap1* functions in social behavior.

Behavioral analyses of the *adap1* mutants suggest the *adap1* gene is involved in the regulation of approach behavior to visual cues from conspecifics. Interestingly, *adap1* KO mice possess social behavior impairments that show a trend toward increased exploratory behavior toward novel objects (Szatmari et al., 2021). Recent accumulating evidence indicates that *adap1* is an Arf6 GTPase‐activating protein and that the loss of *adap1* can activate Arf6 (Szatmari et al., 2021; Venkateswarlu et al., 2004), which can lead to the downregulation of AMPA receptors (Myers et al., 2012; Scholz et al., 2010). AMPA receptor signaling in the ventral tegmental area has been associated with exploratory behavior toward novel stimuli (Bariselli et al., 2018). These findings suggested that increased activation of Arf6 may be involved in the increased exploratory behavior observed in the *adap1* mutants through dysregulation of AMPA receptor signaling. Moreover, genetic mutations in human *adap1* have been associated with neurodevelopmental disorders in patients (Coe et al., 2019; Mastrangelo et al., 2022). Further functional analyses of zebrafish *adap1* mutants in social behaviors will be useful to understand the pathology of neural disorders disrupted the *adap1* gene.

In summary, the *adap1* gene is expressed in the forebrain, midbrain, and hindbrain of zebrafish during early embryogenesis. Zebrafish *adap1* mutants can survive without obvious morphological defects, and adult *adap1* mutants exhibit differences in approach behavior to visual cues from conspecifics, raising the possibility that the *adap1* gene is involved in the etiology of neurodevelopmental disorders.

## FUNDING INFORMATION

This work was supported by the Japan Agency for Medical Research and Development (reference 21ek0109484, 22ek0109484 and 23ek0109484) and Mie University Graduate School of Medicine (2023).

## CONFLICT OF INTEREST STATEMENT

The authors have declared that no competing interests exist.

## Supporting information


**Data S1.** Supporting Information.

**Movie S1.** Spontaneous coiling of wild‐type embryo at 19 hpf.

**Movie S2.** Spontaneous coiling of the *adap1* ^*uy70/uy70*^ mutant at 19 hpf.

**Movie S3.** Spontaneous coiling of the *adap1* ^*uy71/uy71*^ mutant at 19 hpf.

**Movie S4.** Touch response of wild‐type embryo at 28 hpf.

**Movie S5.** Touch response of the *adap1* ^*uy70/uy70*^ mutant at 28 hpf.

**Movie S6.** Touch response of the *adap1* ^*uy71/uy71*^ mutant at 28 hpf.

**Movie S7.** Swimming of adult wild‐type fish.

**Movie S8.** Swimming of adult *adap1* ^*uy70/uy70*^ mutant fish.

**Movie S9.** Swimming of adult *adap1* ^*uy71/uy71*^ mutant fish.
