# Supplementary material for: Establishment and characterization of adap1 ‐deficient zebrafish
Source: Dev Growth Differ. 2025 Mar 15;67(3):165–73. doi: 10.1111/dgd.70004 (PMC11997736; doi:10.1111/dgd.70004)
Supplement: Supplementary file 1 — Data S1. Supporting Information. [file DGD-67-165-s009.docx]

**Establishment and characterization of *adap1*-deficient zebrafish**

Atsuo Kawahara^1#^, Sakyo Yasojima^2#^, Junko Koiwa^2#^, Saori Fujimaki^1^, Hiroaki Ito^2^, Mamiko Yamada^3^, Kenjiro Kosaki^3^, Yuhei Nishimura^2^

**Supplemental Information**

ADAP1 MFQFVFSRVYCINPAR-----RKWKEFEKMLGCAEEGHASLGRDPDWASYTLGVFICLSC 55

Adap1 --------MMENEPERSSGALRDLRQRPGNQSCADC----GAAEPDWVCVNLGVFVCQGC 48

ADAP1 SGIHRNIPQVSKVKSVRLDAWEEAQVEFMASHGNDAARARFESKVPSFYYRPTPSDCQLL 115

Adap1 SLIHRSVFSLCGVKSVLQDSFEDSEIEFISSMGNEAAKAKYEQQVPPFYFRPSHTDCRIL 108

ADAP1 REQWIRAKYERQEFIYPEKQEPYSAGYREGFLWKRGRDNGQFLSRKFVLTEREGALKYFN 175

Adap1 REQWIRAKYERQEFIHIERQEPYSAGYREGFLWKRGRDNGQFLSRKFILSEREGALKYFN 168

ADAP1 RNDAKEPKAVMKIEHLNATFQPAKIGHPHGLQVTYLKDNSTRNIFIYHEDGKEIVDWFNA 235

Adap1 KQDAREPKAIMRIETLNAAFQPAKIGNPCGLQITYLKDNSTRNIFVYHEDSKEMVDWFTA 228

ADAP1 LRAARFHYLQVAFPGASDADLVPKLSRNYLKEGYMEKTGPKQTEGFRKRWFTMDDRRLMY 295

Adap1 IRAARFHYQKVAFPGANDEDLVPRLTRNFTKEGFMQKTGPRHTEGFKKRWFTMDDRRLMY 288

ADAP1 FKDPLDAFARGEVFIGSKESGYTVLHGFPPSTQGHHWPHGITIVTPDRKFLFACETESDQ 355

Adap1 FKDPLDAYALGEVFIGSKENRYTVLSGLPPSTQGYHWKYGITIETPDRKFLFACETEAEQ 348

ADAP1 REWVAAFQKAVDRPMLPQEYAVEAHFKHKP 385

Adap1 KDWIAAFQRVVNRPMMPQEYAVEAHFKHKP 378

**Figure S1 Amino acid sequence alignment of human ADAP1 and zebrafish Adap1.**

Amino acid sequences of human ADAP1 (upper) and zebrafish Adap1 (lower) were aligned using the Crustal Omega program, and conserved amino acids are indicated in red.

**
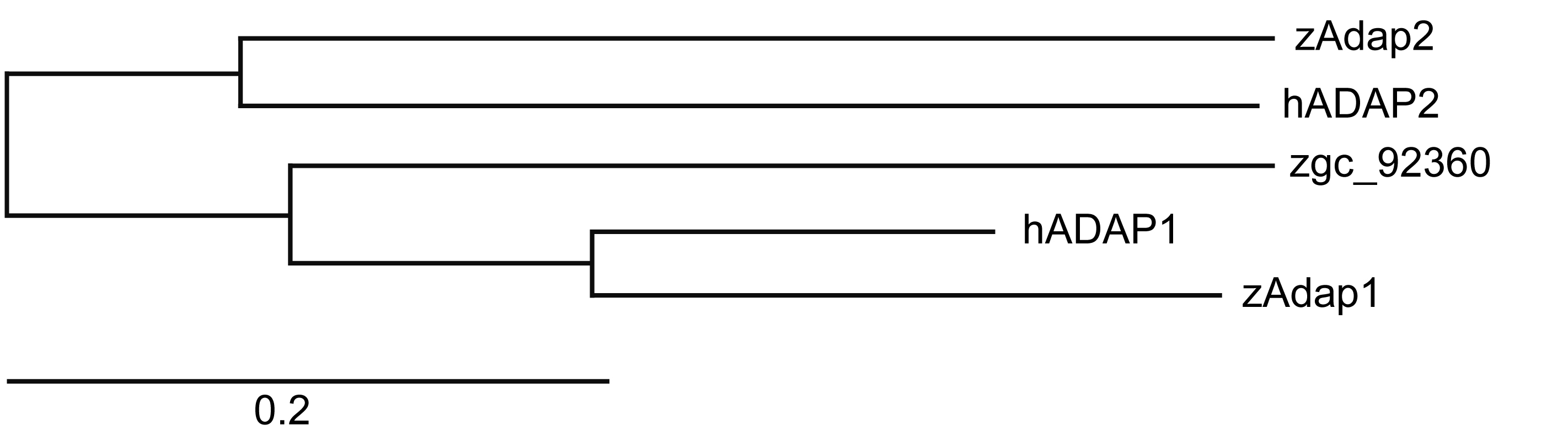
**

**Figure S2 Phylogenic analysis based on zebrafish and human Adap proteins**

Amino acid sequences of zebrafish and human Adap proteins were aligned with MAFFT and the tree was constructed with neighbor joining algorithm. Scale bar indicates branch length.

Adap1 wild-type

MMENEPERSSGALRDLRQRPGNQSCADCGAAEPDWVCVNLGVFVCQGCSLIHRSVFSLCGVKSVLQDSFEDSEIEFISSMGNEAAKAKYEQQVPPFYFRPSHTDCRILREQWIRAKYERQEFIHIERQEPYSAGYREGFLWKRGRDNGQFLSRKFILSEREGALKYFNKQDAREPKAIMRIETLNAAFQPAKIGNPCGLQITYLKDNSTRNIFVYHEDSKEMVDWFTAIRAARFHYQKVAFPGANDEDLVPRLTRNFTKEGFMQKTGPRHTEGFKKRWFTMDDRRLMYFKDPLDAYALGEVFIGSKENRYTVLSGLPPSTQGYHWKYGITIETPDRKFLFACETEAEQKDWIAAFQRVVNRPMMPQEYAVEAHFKHKP

Adap1-yu70 Mutant

MMENEPERSSGALRDLRQRPGNQSCADCGAAEPDWVCVNLGVFVCQGCSLIHRSVFSLCGVKSVLQDSFEDSEIEFISSMGNEAAKAKYEQQVPPFYFRPSHTDCRILREQWIRAKYERQEFIHIDRSRTPQVRNATGRVFCGSVDGTTDSS

Adap1-uy71 Mutant

MMENEPERSSGALRDLRQRPGNQSCADCGAAEPDWVCVNLGVFVCQGCSLIHRSVFSLCGVKSVLQDSFEDSEIEFISSMGNEAAKAKYEQQVPPFYFRPSHTDCRILREQWIRAKYEQEFIHIDRSRTPQATGRVFCGSVDGTTDSS

**Figure S3 Amino acid sequences of wild-type and mutant zebrafish Adap1 proteins.**

Red letters indicate the ArfGAP domain. Green and blue letters indicate the PH1 and PH2 domains, respectively. The purple letters indicate missense amino acids.

**
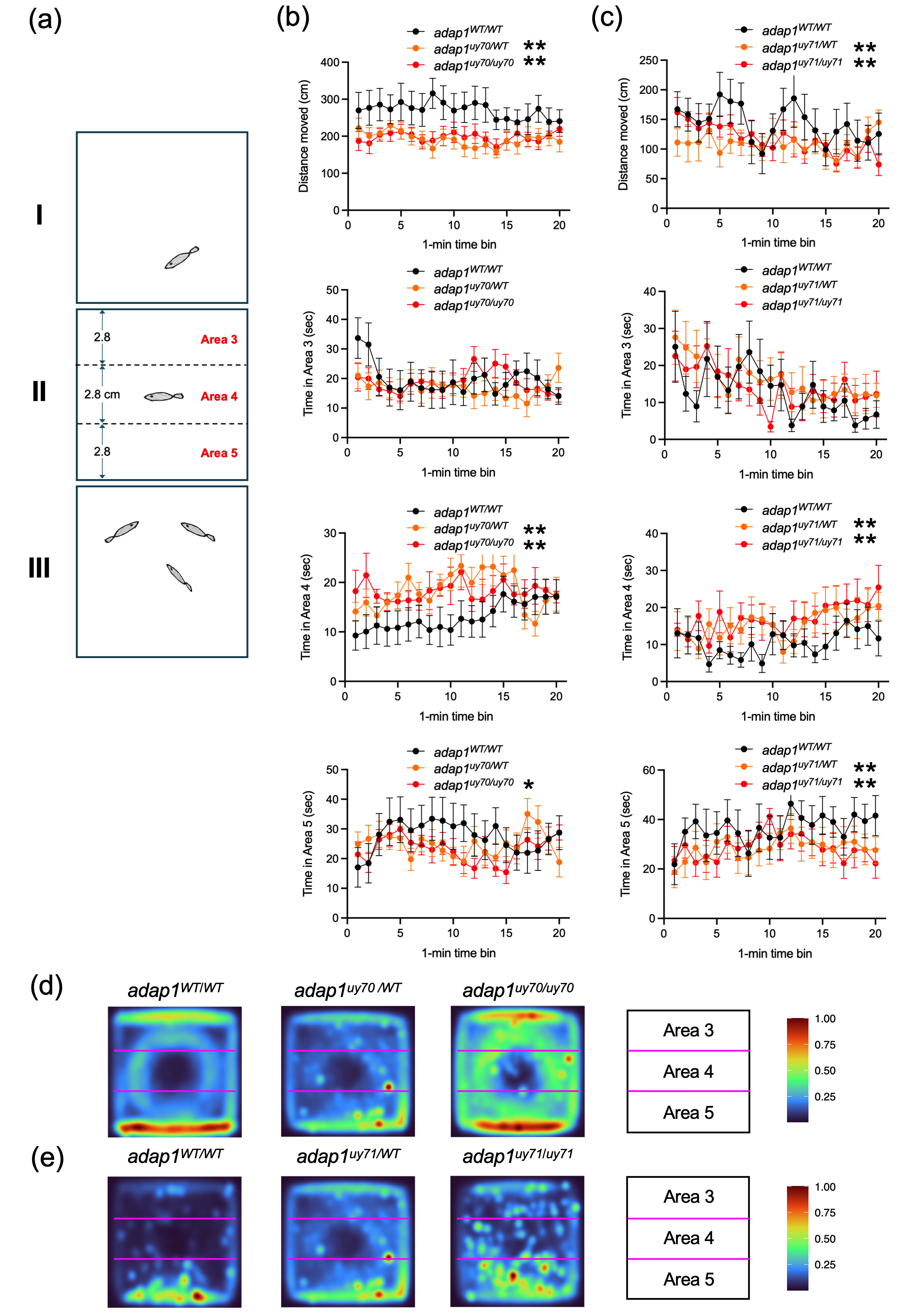
**

**Figure S4 Social behavior analysis of wild-type and *adap1* mutants in the adult stage using familiar conspecifics.**

(a) Schematic representation of the social behavior test used in this study. Areas 3 and 5 were set as the regions near the stimulus exploration. One and three familiar conspecifics were placed in Chamber I and III, respectively. (b and c) The distance moved by zebrafish in Chamber II and the time in the areas where zebrafish in Chamber II were located for each minute. The *adap1^uy70/WT^*, *adap1^uy70/uy70^*, *adap1^uy71/WT^*, and *adap1^uy71/uy71^* swam shorter in Chamber II. The *adap1^uy70/WT^*, *adap1^uy70/uy70^*, *adap1^uy71/WT^*, and *adap1^uy71/uy71^* spent more time in Area 4. The *adap1^uy70/uy70^*, *adap1^uy71/WT^*, and *adap1^uy71/uy71^* spent less time in Area 5. The numbers of zebrafish are Ν=8 for WT/WT, Ν=14 for WT/*uy70*, Ν=10 for *uy70*/*uy70*, Ν=7 for WT/WT, Ν=13 for WT/*uy71*, Ν=11 for *uy71*/*uy71*. *P<0.05, **P<0.01. (d and e) Heatmap of the behavior of wild-type and *adap1* mutants. The numbers of zebrafish in each group are same in (b and c). A high number of color indicators indicates that zebrafish were frequently located at that position during the 20 min test period.

**Supplemental Tables**

**Table S1 Targeted genomic sequences.**

Targeted genomic sequences for CRISPR–Cas9

| Target | Sequence (5' to 3') |
| --- | --- |
| *adap1*-1 | CAGAGCCAAATACGAGCGACAGG |
| *adap1-2* | GTTTATACACATCGAGAGACAGG |

PAM sequences are underlined.

**Table S2 PCR primers used in this study.**

| Primer name | Sequence (5' to 3') |
| --- | --- |
| adap1-HMA-F1 | TGCGGGAGCAGTGGATCAGAG |
| adap1-HMA-R1 | GTTTCTGACCTGCGGAGTACG |

**Supplemental Movie S1: Spontaneous coiling of wild-type embryo at 19 hpf.**

**Supplemental Movie S2: Spontaneous coiling of the *adap1^uy70/uy70^* mutant at 19 hpf.**

**Supplemental Movie S3: Spontaneous coiling of the *adap1^uy71/uy71^* mutant at 19 hpf.**

**Supplemental Movie S4: Touch response of wild-type embryo at 28 hpf.**

**Supplemental Movie S5: Touch response of the *adap1^uy70/uy70^* mutant at 28 hpf.**

**Supplemental Movie S6: Touch response of the *adap1^uy71/uy71^* mutant at 28 hpf.**

**Supplemental Movie S7: Swimming of adult wild-type fish.**

**Supplemental Movie S8: Swimming of adult *adap1^uy70/uy70^* mutant fish.**

**Supplemental Movie S9: Swimming of adult *adap1^uy71/uy71^* mutant fish.**
